# Supplementary figures and images for: Lipids associated with plant-bacteria interaction identified using a metabolomics approach in an Arabidopsis thaliana model
Source: PeerJ. 2022 Apr 27;10:e13293. doi: 10.7717/peerj.13293 (PMC9055996; doi:10.7717/peerj.13293)

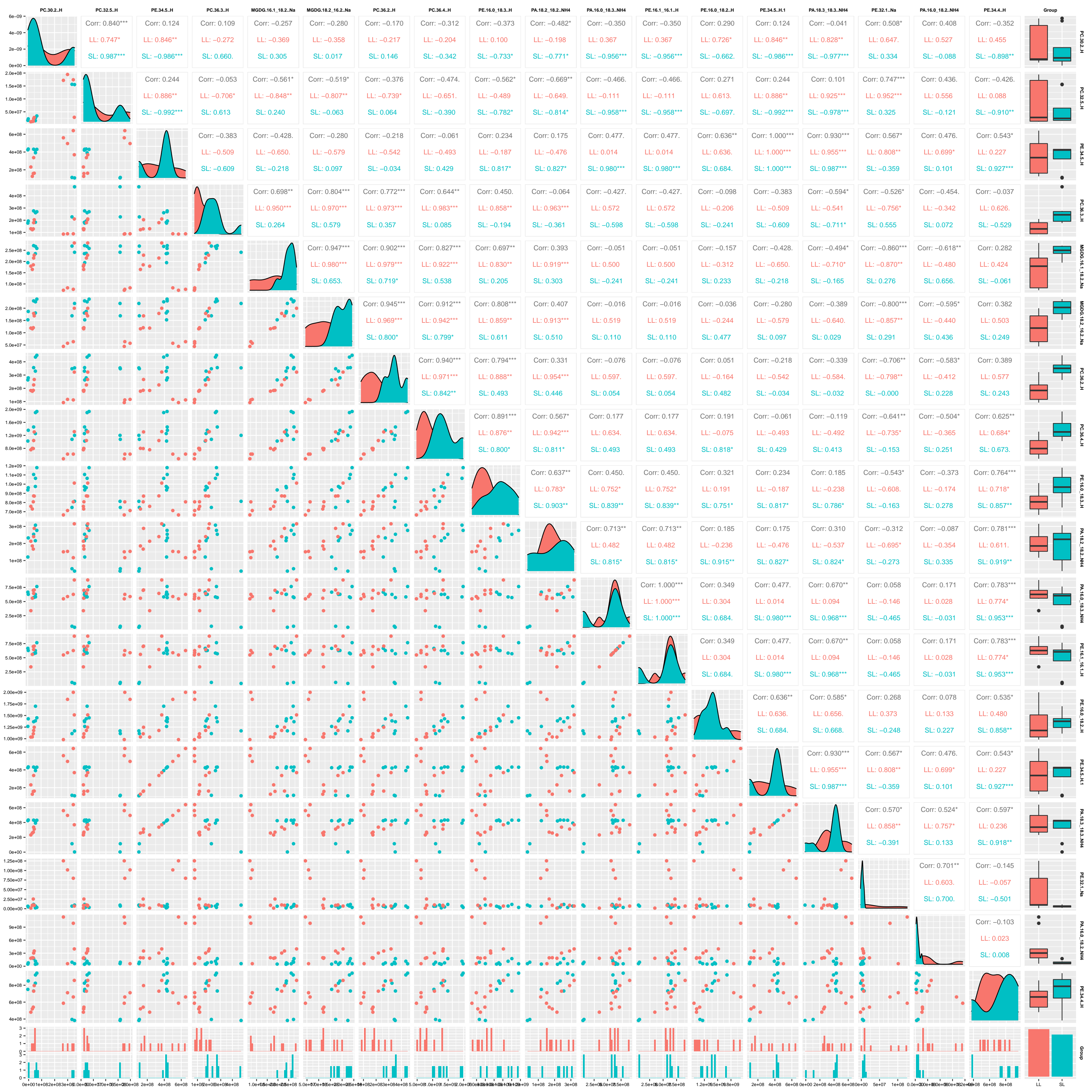

Supplement: Supplemental Information 2 [file peerj-10-13293-s002.pdf]
