# Supplementary material for: Lipids associated with plant-bacteria interaction identified using a metabolomics approach in an Arabidopsis thaliana model
Source: PeerJ. 2022 Apr 27;10:e13293. doi: 10.7717/peerj.13293 (PMC9055996; doi:10.7717/peerj.13293)

You can access the following link to get the raw data:

https://www.iprox.cn/page/PSV023.html;?url=1629267250633J4Ws. Password: qfHV. There is no need for a username and password. The steps are as follows:


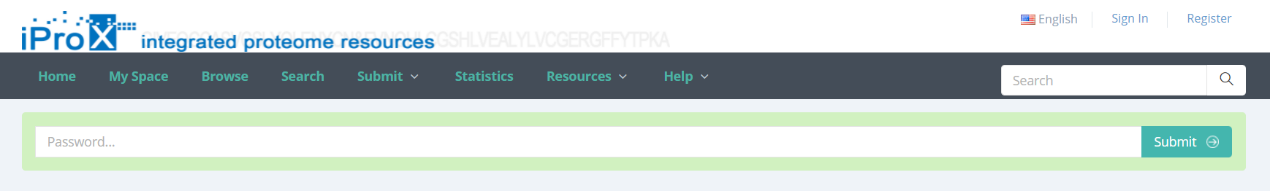


There don't need to click "sign in"


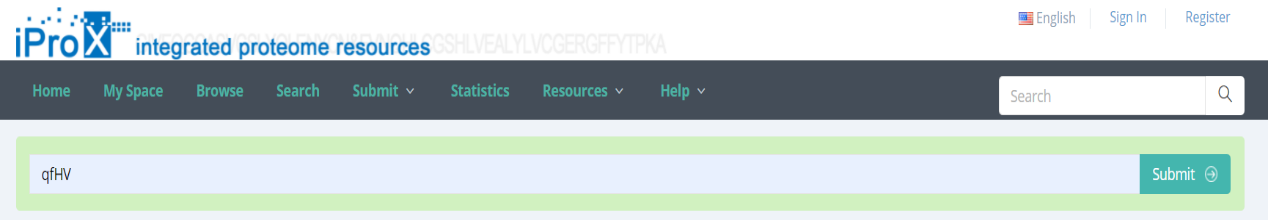


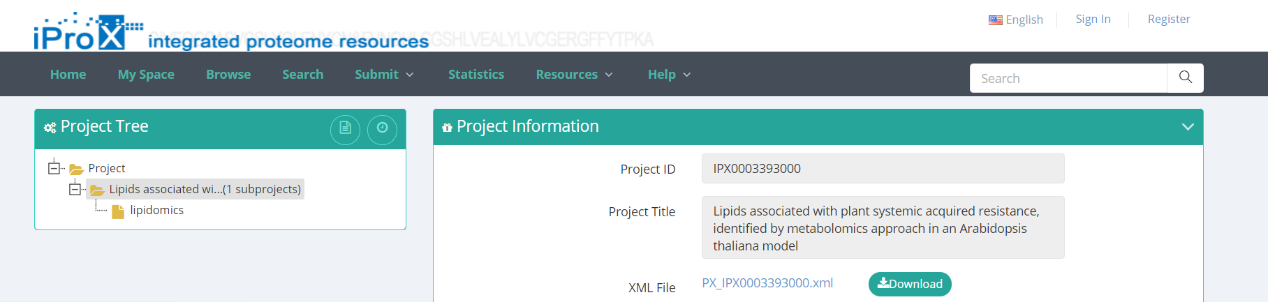

Supplement: Supplemental Information 4 [file peerj-10-13293-s004.docx]
